# A review of clinical models for the evaluation of human TB vaccines

**DOI:** 10.1080/21645515.2015.1134407

**Published:** 2016-01-25

**Authors:** Matthew K. O'Shea, Helen McShane

**Affiliations:** The Jenner Institute, University of Oxford, Oxford, UK

**Keywords:** BCG, clinical models, clinical trials, MVA85A, *Mycobacterium tuberculosis*, tuberculosis, vaccine

## Abstract

While much progress has been made in the fight against the scourge of tuberculosis (TB), we are still some way from reaching the ambitious targets of eliminating it as a global public health problem by the mid twenty-first century. A new and effective vaccine that protects against pulmonary TB disease will be an essential element of any control strategy. Over a dozen vaccines are currently in development, but recent efficacy trial data from one of the most advanced candidates have been disappointing. Limitations of current preclinical animal models exist, together with a lack of a complete understanding of host immunity to TB or robust correlates of disease risk and protection. Therefore, in the context of such obstacles, we discuss the lessons identified from recent efficacy trials, current concepts of biomarkers and correlates of protection, the potential of innovative clinical models such as human challenge and conducting trials in high-incidence settings to evaluate TB vaccines in humans, and the use of systems vaccinology and novel technologies including transcriptomics and metabolomics, that may facilitate their utility.

## Introduction

In the 1990s the World Health Organization (WHO) set targets to identify 70% and cure 85% of TB cases, which were reached in many countries by 2005.^1,2^ Subsequently, targets to achieve a 50% reduction in the global mortality and prevalence of TB disease by 2015 were established by the Stop TB Partnership in 2000.^3^ It has been estimated that in 2013 there were 9 million new cases and 1.5 million deaths attributed to TB, representing reductions in TB mortality and prevalence of 45% and 41%, respectively.^4^

While this progress is significant, it is insufficient. It represents a reduction in the global TB incidence of approximately 1.5% per year over recent years.^4^ However, to achieve the WHO target to eliminate TB as a global public health problem by 2050 (<1 case per million per year), a 1,000-fold reduction in global TB incidence over the next 35 years is required, corresponding to an unprecedented 20% reduction per year.^3,5^

Until recently, the main focus of TB control and elimination strategies has been the prompt diagnosis and effective treatment of individuals with active disease to interrupt transmission. This approach is important, however even if transmission were interrupted completely and instantly in 2015, reactivation of established, and relapse of persisting, *Mycobacterium tuberculosis (M. tb)* infection would still cause an estimated >100 cases per million population in 2050.^5^ An effective pre-exposure vaccine to protect against *M. tb* infection would not only be the most cost effective approach of control, but would also be a crucial component of any strategy to eliminate the global burden of TB.^6,7^ In addition, there is an increasing realization that the management of latently infected individuals, which represents a huge reservoir of potential new disease and thus infectiousness, will also be required to reduce TB disease. Modeling suggests that the effective treatment of latent infection with a drug or a vaccine (and likely both) would reduce TB incidence significantly.^8^

## TB vaccines – the old and the new

Bacille Calmette-Guerin (BCG), an attenuated strain of *M. bovis*, is the only licensed vaccine for TB.^9^ It has been part of the WHO Expanded Programme on Immunisation (EPI) since the early 1970s and is the most widely used vaccine in history, with over 4 billion doses administered to date. The WHO currently recommends that a single dose of BCG be given to neonates or as soon as possible after birth in countries with a high prevalence of TB.^10^ Low-burden countries may choose to limit BCG vaccination to neonates and infants of recognized high-risk groups for TB or to tuberculin-negative older children. BCG vaccination is also recommended for unvaccinated, tuberculin-negative persons in non-endemic areas who are exposed to multi-drug resistant *M. tb*.

BCG has been shown to be effective at preventing disseminated TB disease, such as miliary and meningeal TB, in children.^11^ In addition, BCG vaccination is thought to have non-specific effects and there is evidence that it correlates with a reduction in general infant mortality.^12,13^ While generally safe, it is not recommended for use in immunocompromised individuals due to concerns over the possible development of disseminated BCG disease.^14,15^

However, it is considered that BCG vaccination has had little impact on the overall incidence of TB.^16^ The efficacy of BCG is highly variable, ranging from 0–80% in different settings, vaccine-induced immunity may wane with time, and it fails to confer adequate protection against pulmonary disease, particularly among adolescents and young adults in high-endemic regions.^17-21^ This is a significant problem as this population continues to propagate the TB epidemic; the incidence of latent TB infection (LTBI) reaches 60–70% in adults aged over 25 years in most affected areas.^22^ There is therefore an urgent need to develop novel, effective, TB vaccines.

Vaccines against TB may be either prophylactic and/or therapeutic and have the potential to be directed against several stages of *M. tb* infection and disease. Prophylactic vaccines may be administered either pre- or post-exposure to *M. tb* and mathematical modeling suggests that such deployment would most rapidly achieve global control of the TB epidemic.^8^ A pre-exposure vaccine would prevent primary acquisition of *M. tb* infection and would ideally be administered in infancy, prior to infection. A post-exposure vaccine would be administered to adolescents and young adults following infection with *M. tb* to prevent post-primary disease and/or reactivation of latent infection. Finally, a therapeutic vaccine would target individuals with active TB disease as adjunctive therapy to simplify, enhance the efficacy of, and shorten drug treatment. Such vaccines should be effective against both drug-sensitive and resistant strains of *M. tb*.^16,23^ In addition, the ideal vaccine would be safe in all age groups, in patients with HIV infection and would induce long-term and effective immunological memory, abrogating the need for repeated vaccination or boosting.^23^

Current strategies to develop an improved TB vaccination regimen have focused on improving BCG, boosting it, or replacing it with a different vaccine altogether.^24-26^ The fact that many of these strategies attempt to exploit the immunity induced by priming with BCG is logistically pragmatic as the majority of the target population has been vaccinated with BCG in childhood as part of EPI. TB vaccines can be broadly classified as either subunit or whole, live-attenuated, mycobacterial vaccines, and at present there are 16 candidate vaccines in active clinical evaluation (Table 1).^27^ A detailed description of these has recently been undertaken and is beyond the scope of this article, however valuable lessons may be drawn from reviewing one of the most advanced of the candidate TB vaccines, MVA85A.^28,29^

**Table 1.** Summary of TB vaccines currently under clinical assessment (adapted from^27^).

Strategy	Vaccine candidate	Vaccine type	Phase	Sponsor
Prime	MTBVAC	Live genetically attenuated *M. tb*	IIa	University of Zaragoza; Biofabri; Tuberculosis Vaccine Initiative (TBVI)
	VPM1002	Live recombinant BCG	IIa	Serum Institute of India; Vakzine Projekt Management; TBVI; Max Planck Institute for Infection Biology
Prime-boost	M72/AS01	Protein/adjuvant	IIb	GlaxoSmithKline; Aeras
	Hybrid 4 + IC31	Protein/adjuvant	IIa	Statens Serum Institut (SSI); Sanofi Pasteur; Valneva; Aeras
	Hybrid 56 + IC31	Protein/adjuvant	IIa	SSI; Valneva; Aeras
	Hybrid 1 + IC31	Protein/adjuvant	IIa	SSI; Valneva
	Ad5Ag85A	Viral vector	I	McMaster University; CanSino
	Crucell Ad35 + MVA85A	Viral vector	I	Crucell; Oxford University; Aeras
	ChAdOx1.85A + MVA85A	Viral vector	I	Oxford University
	Dar-901	Whole-cell *M. obuense*	I	Dartmouth University; Aeras
	MVA85A (aerosol)	Viral vector	I	Oxford University
	MVA85A-IMX313	Viral vector	I	Oxford University; Imaxio
	ID93 + GLA-SE	Protein/adjuvant	I	Infectious Disease Research Institute; Aeras
	TB/FLU-04L	Viral vector	I	Research Institute for Biological Safety Problems
Immunotherapeutic	*M. vaccae*	Whole-cell *M. vaccae*	III	AnHui Longcom
	RUTI	Fragmented *M. tb*	IIa	Archivel Farma

## Lessons from recent efficacy trials

MVA85A is a recombinant strain of Modified Vaccinia virus Ankara expressing the conserved *M. tb* antigen 85A.^30^ While still important in terms of immunogenicity, recent evidence suggests that antigen 85A is less immunodominant than previously thought.^31^ MVA85A was developed for a heterologous prime-boost strategy, to be administrated following BCG vaccination to augment antigen-specific T cells. It has recently completed the largest infant phase IIb efficacy trial since the introduction of BCG over 90 years ago in a BCG-prime, MVA85A-boost regimen, and the disappointing results have highlighted the significant challenges in the field of TB vaccine development and testing.^32^

Extensive preclinical studies in animal models (mice, guinea pigs and non-human primates) demonstrated that boosting of BCG with MVA85A could improve protection against mycobacterial challenge, although not consistently in every challenge experiment.^33-36^ There are several limitations to these models which are discussed below and extensively reviewed elsewhere.^37^

Several human phase I/IIa studies in both high and low disease burden settings among adults, adolescents, children and infants showed MVA85A to be safe and immunogenic (the two endpoints tested in such trials).^38-48^ Among healthy, *M. tb*-infected or HIV-infected individuals, MVA85A induced antigen-specific Th1 and Th17 cells, which are both considered important in protection against *M. tb*.^30,43,46,47^

Recently, almost 2800 healthy, HIV-negative, BCG-vaccinated South African infants (4–6 months old) were randomized to receive either MVA85A or placebo. MVA85A was well tolerated but induced only modest antigen-specific T cell responses (several-fold lower than those seen in UK adults) and did not confer any additional protection over BCG alone to *M. tb* infection (vaccine efficacy was −3.8% and 17.3% against *M. tb* infection and disease, respectively).^32^ These outcomes were clearly in contrast to the earlier encouraging preclinical results.

A further phase IIb trial assessing the efficacy of MVA85A in over 650 healthy adults infected with HIV in South Africa and Senegal has recently been reported.^49^ MVA85A was safe and immunogenic, inducing significant increases in antigen-specific T cell responses which were primarily monofunctional interferon gamma (IFN-γ) and tumor necrosis factor α (TNF-α)-producing CD4+ T cells. However, there was no efficacy against *M. tb* infection or disease in the MVA85A group when compared to placebo (vaccine efficacy was 11.7% and 32.8% against *M. tb* infection and disease, respectively).

Despite these outcomes, the advanced MVA85A trials have been extremely important. Firstly, they demonstrate that it is feasible to conduct large-scale clinical efficacy trials of vaccines against TB in high-burden settings and in the target population. Secondly, the results raise several fundamental questions relevant to the whole field, which has stimulated debate and generated innovative proposals for the evaluation of TB vaccine candidates in the future.

It is now evident that the variable and modest efficacy seen in preclinical animal models was not able to predict protection in BCG-vaccinated infants or HIV-infected adults. Several reasons have been suggested to account for the disparity between the animal data and the outcomes of human efficacy trials.^37^

Species differences that may influence the predictive ability of animal models exist. The manifestations of *M. tb* infection and disease are different between species and immune responses to vaccination are more variable in humans.^19,50^ For example, the structure and heterogeneity of murine granulomas do not mimic those seen in humans infected with *M. tb* and there are no simple animal models of latent *M. tb* infection that easily represent the human situation except potentially a non-human primate model.^51^ There is also genetic immunological variation between species (e.g. the absence of most CD1 subtypes in mice), with implications on correlating immune responses with those seen in humans.^52^ Furthermore, experimental animal models generally use adult animals and while the target population for some candidate vaccines is human adults, many are designed for use in infants and adolescents. Immunogenicity studies are needed to assess different immune responses at various ages.

Secondly, there are significant differences in the nature of exposure between animal challenge experiments and natural infection in humans. In the former, laboratory strains of *M. tb* are used in a single exposure via a variety of challenge routes and at much higher inocula than that seen in natural infection. However, humans are likely to experience multiple low dose exposures of clinical strains, with the establishment of infection following one or several of these exposures (or, indeed, not at all).^53^ Recent evidence from a study assessing the protection of BCG and a novel candidate vaccine against newly emerging, mostly highly virulent, strains of *M. tb* has highlighted the importance of the fitness of prevalent strains of *M. tb* at clinical trial sites when trying to show vaccine efficacy.^54^

There is, therefore, a need to develop experimental models of infection more comparable to natural challenge.

Thirdly, there are fundamental differences in study power, definitions and endpoints. The MVA85A efficacy trial was powered to detect a 60% improvement over BCG, however the magnitude of candidate vaccine efficacy in animal models was much lower than this and would not have been detected in clinical trials.^32,37^ Also, the definition of protection varies between human and animal models. In animals, protective efficacy of a candidate vaccine is assessed in terms of improvement in the extent of disease using markers such as organ bacillary load, severity of pathology or time to death. In contrast, human trials define efficacy as the prevention of disease. Clearly, there is a fundamental difference between these endpoints, which lack intuitive correlation. It has been suggested that changing animal trial endpoints from TB disease reduction to disease prevention should be considered and, still further, possibly establishing animal challenge models to show prevention of *M. tb* infection rather than protection from disease, which may be a more feasible endpoint in human efficacy trials.^37^ However, limitations of this approach include the need to use much larger numbers of animals, and the lack of validated biomarkers of *M. tb* infection to correlate parameters in animal models with human efficacy data. As discussed later, while to date the focus of human efficacy trials has been prevention of disease, the low number of endpoints requires large clinical trials which are both expensive and time consuming. Recently there has been renewed interest in using prevention of infection as a measure of candidate vaccine efficacy which would provide more endpoints but presupposes that the underlying immune mechanisms of *M. tb* infection and disease are similar and highlights the limitations of diagnostics and the definition of *M. tb* infection.^55^

Finally, there are several other aspects of variation in human clinical trial settings that contrast with the laboratory, such as nutritional status and diet, exposure to non-tuberculous mycobacteria (NTM), helminth infection and the effect of host genetic heterogeneity on susceptibility. For example, several studies have shown poor BCG vaccine efficacy in human populations with high levels of prior exposure to environmental (NTM).^56-59^ A recent systematic review of randomized controlled trials found that the absence of sensitization with NTM was associated with higher efficacy of BCG against pulmonary (and also possibly miliary and meningeal) TB.^60^ It has been suggested that pre-existing immunity to NTM results in either ‘blocking’ the effects of BCG vaccination by inhibiting the replication of BCG and preventing the induction of protective immune responses or ‘masking’ the effects as BCG is unable to further boost background immunity induced by NTM. In addition, chronic helminth infections may also have an modulatory effect on vaccine efficacy, causing a shift toward Th2-type immunity, impaired antigen-specific and Th1-type responses and the induction of regulatory T cells producing transforming growth factor-β and other inhibitory cytokines which suppress pro-inflammatory cytokines. These responses have been associated with reduced efficacy of BCG in endemic settings.^61-64^

Fundamental questions that remain are what is the magnitude of improvement in animal challenge models needed to predict the significant improvement in protection required in humans and which immunological parameters have the greatest predictive power for vaccine efficacy? Recent efficacy trial data have highlighted the need to establish biomarkers that correlate with vaccine-induced protection against TB disease as, without such biomarkers, the only way to safely assess vaccine efficacy is by large trials with the diagnosis of TB disease as the endpoint.

## Biomarkers and correlates of protection – what should we look for, how and in whom?

### Current concepts

Immune correlates of vaccine-induced protection are biomarkers that reliably predict the level of protective efficacy induced by a vaccine. A biomarker is a unique indicator of a biological process and a biosignature is a combination of independent biomarkers which markedly increases the power of an individual marker.^65,66^ Biomarkers for vaccines are typically identified by assessing differences in immune parameters between vaccinated individuals who are protected and unvaccinated, unprotected, control groups.^67^ At present there are no validated correlates to reliably assess the efficacy of candidate TB vaccines. Moreover, host biomarkers are urgently needed to improve TB diagnostics and for the development of more effective and shorter treatment regimens. The perfect biomarker or biosignature for TB would differentiate between patients with active disease and latent infection, return to baseline levels following successful treatment, reproducibly predict clinical outcomes, predict vaccine efficacy and provide endpoints for clinical trials.^67^ As such, robust biomarkers would have significant utility as surrogate endpoints rather than relying on clinical endpoints. However, based on our current understanding and knowledge of the biological and immunological responses that underlie discrete states of TB immunopathogenesis, it is unlikely that such a perfect biomarker exists.

The lack of a correlate of protection is a significant obstacle in TB vaccine development and persists due to our incomplete understanding of the natural infection and mechanisms contributing to host immunity to TB. To date, the most common parameters measured to assess TB vaccine immunogenicity are those considered important for protection against infection or disease. These have been determined through observational studies of mycobacterial susceptibility in humans and experimental animal models. The importance of IFN-γ production by T cells is demonstrated by the significant increases in rates of *M. tb* in conditions of immune deficiency. These may be primary (e.g., the syndromes of Mendelian susceptibility to mycobacterial diseases, MSMD) or acquired (e.g. HIV infection) and reduce the number and/or function of CD4+ T cells or impair IFN-γ signaling.^68-70^ As such, the primary immunological readout of TB vaccine studies is antigen-specific IFN-γ production by T cells, typically using ELISpot assays.^67^ In addition, other Th1 cytokines such as TNF-α and frequencies of polyfunctional CD4+ T cells, determined by multi-parametric flow cytometry, are also thought to be important.^67,71^ However, there is clearly a disparity between those immunological responses stimulated by vaccination (correlates of immunogenicity) and those associated with protection from TB disease (correlates of protection).^72^ Several studies have shown that while significant increases in antigen-specific IFN-γ secretion and changes in polyfunctional T cell profiles may be induced by vaccination, these responses do not correlate with protection against *M. tb*.^32,40,73-75^

Thus far, the immune responses considered essential for protection may well be necessary but they are not sufficient and as such these parameters do not have utility as correlates of risk or protection in TB vaccine efficacy trials. Other elements thought to have a role in protection that warrant further investigation include IL17-producing Th-17 cells, regulatory CD4+ T cells, CD8+ T cells, γδ cells, natural killer cells and components of innate immunity. The role of B cells also remains to be defined.^67,71^ It is likely that rather than a simple effector or memory output (such as that for the serogroup C meningococcal vaccine), multiple factors deployed in a coordinated and balanced immune response will be crucial for effective protection and therefore should be assessed in concert.^76^

Characterizing naturally induced protection in *M. tb* infection among household contacts exposed to patients with pulmonary disease could be used to identify biomarkers that correlate with protective immunity.^71^ Such contacts exhibit diverse immune responses that would be suitable to study using systems vaccinology and would improve our understanding of the spectrum of *M. tb* immunopathology. For example, it is hypothesized that, despite significant exposure to *M. tb*, some individuals have no evidence of immune sensitization which is likely due to inherent resistance or the elimination of infection through an effective innate immune response or non-primed adaptive immunity. In contrast, latently infected, asymptomatic individuals, probably exist on a spectrum of *M. tb* infection and exhibit immunological evidence of T cell priming and persisting quiescent infection which is controlled by the acquired immune response.^77,78^ Within this heterogeneous group the host-pathogen relationship is highly dynamic and some individuals may eventually effectively eliminate the infection, others will maintain persistent, life-long infection, while another group will develop subclinical disease and progress to primary active disease or reactivation TB.^77,79,80^ Household contacts therefore define a range of immune phenotypes that could help in characterizing correlates of risk and protection with application to TB vaccine development. Studies of this group are also relevant as individuals with LTBI are a potential target population for vaccination.

### The potential and pitfalls of systems vaccinology

One potential method to identify biosignatures of protective immunity to TB is to use high-throughput ‘omics’ technologies in a ‘systems vaccinology’ approach.^72,81–84^ This approach can be used to study the mechanisms of vaccine-induced immunity by assessing the dynamics and interactions of multiple components of the immune system through iterative cycles of perturbations and high-throughput biology. It is exemplified by early studies with the yellow fever vaccine, YF-17D, in which systems biology was used to identify the mediators and predictors of the immune response following vaccination.^85,86^ Similar approaches have now been applied to several other vaccines.^72,87^

Systems vaccinology is based on the same principles as systems biology. Following vaccination, perturbations of the immune system are profiled by using high-throughput techniques on biological samples (e.g., DNA and RNA sequencing, transcriptomic microarrays, proteomics and metabolomics). Data derived from these multiple platforms are then integrated with those obtained from assays routinely used in vaccinology (e.g. ELISpot and flow cytometry). Mathematical models are subsequently created from a variety of modeling frameworks to describe and/or predict the vaccine-induced immune responses observed.^88^ Such biosignatures must then be validated using independent sets of samples.^72,89^

A significant benefit to using ‘omic’ technologies is that they offer an unbiased, hypothesis-generating approach from which findings may be subsequently investigated by more targeted experiments. As described, we still do not fully understand the basis of host immunity to *M. tb* or the protection afforded by vaccination, and immune responses are heterogeneous and controlled by several levels of regulation. While targeted approaches assessing specific biomarkers such as cytokine production or the characterization of immune cell phenotypes in antigen-stimulated samples (e.g., whole blood or peripheral blood mononuclear cells) have advanced our knowledge, they are insufficient. Moreover, peripheral blood samples, though easily obtainable, may not represent the complete immunological milieu at the principal interface between host and mycobacteria - the pulmonary granuloma. This structure provides the morphological architecture for regional immune processes which are central to outcome in TB.^90^ Untargeted approaches using more appropriate material, such as bronchoalveolar lavage samples and lung tissue, may yield greater insights and contribute to filling some of the gaps in our knowledge.

Genome-wide differential gene expression studies, typically microarray transcriptomics, represent the archetypal ‘omic’ approach. Several studies have identified gene signatures that discriminate individuals with TB from healthy controls. Increased IFN-α/β signaling, pro-inflammatory signaling through the Janus kinase pathway, and differential expression of Fc-γ receptors, innate immune-related genes and gene clusters involved in apoptosis and natural killer cell activity have all been described.^91-101^ Changes in the transcriptome during treatment, indicative of modulation of humoral responses, have also been reported.^102^ Interestingly, a recent combined ‘meta-like’ analysis of all transcriptomic data reported in human TB pathogenesis showed a myeloid-derived inflammatory signature to be of particular importance.^103^ There are undoubtedly unresolved issues in this area, such as the need to define disease-specific profiles and difficulties in comparing data from studies using different cohorts, experimental techniques and approaches to data analysis.^104^ However, there are also several exciting developments including the increasing availability of affordable RNA sequencing and growing interest in the role of microRNAs (miRNAs) in TB and their potential utility as biomarkers and even as targets for therapeutic intervention.^105-111^

Proteomic profiling of serum samples from TB patients using high-resolution mass spectrometry or protein microarrays has identified several peptides and antibodies that may have diagnostic potential.^112-116^ Most recently, mass spectrometry has also been used to isolate a novel antigen from human TB granulomas.^117^

Finally, metabolic profiling is the identification of small molecular metabolites (e.g. amino acids, lipids, fatty acid, sugars and nucleotides) in clinical samples using high-throughput methods. This approach has shown distinct metabolic biosignatures associated with different TB disease states and responses to treatment in serum, urine, and breath from patients and uninfected controls.^118-121^ Differential profiles have also been reported between different lineages of infecting mycobacteria following anti-TB chemotherapy.^122^

However, there are several challenges to developing systems vaccinology. Integrating large datasets from different techniques is computationally complex and advances in bioinformatics are needed to obtain the greatest value from these high-throughput data.^123^ Recent progress in this area includes the rationalisation of large numbers of genes into smaller, distinct immune modules in which the constituents are highly related and typically expressed in a coordinated way.^124^ This approach is logical as biological processes occur in a modular manner and it affords simpler data analysis and more intuitive interpretation. It has been applied successfully to several diseases and infections (including *M. tb*) and recently to vaccination studies.^91,125–127^

Systems vaccinology is an exciting development and potentially an extremely powerful tool for understanding vaccine-induced immunity and predicting vaccine efficacy. However, it will need further refinement and close collaboration between vaccinologists, immunologists and bioinformaticians if it is to yield truly valuable outputs.

### Alternative approaches to clinical efficacy trials

An alternative, but complementary approach, to testing vaccine efficacy in clinical trials is to identify correlates of protection using human pathogen challenge models. Such models have shown great utility in malaria, influenza, dengue, cholera and typhoid vaccine development.^128-135^ They also have the potential advantage of gating promising vaccine candidates at an early stage, with significant implications on cost and time.

Clearly, it is not possible to challenge volunteers with strains of virulent *M. tb*, however there is increasing interest in using BCG derived from *M. bovis* as a surrogate for *M. tb* infection in a human mycobacterial challenge model. The basis of this model in the hypothesis that an effective TB vaccine, which reduces or prevents *M. tb* replication, should have a similar effect on BCG replication. The benefits of using BCG in this model include that it has significant genetic sequence homology to live *M. bovis* (and therefore *M. tb*), it is a functional, replicating organism that results in limited infection in the immunocompetent host and, crucially, it is safe and licensed for use in humans by intradermal administration.^136^ Optimisation of a BCG human challenge model could be used to establish the clinical parameters for subsequent challenge models using attenuated *M. tb* strains.

A murine model has shown that live BCG can persist in skin for at least 4 weeks and in draining lymph nodes for up to 12 weeks, and that intradermal BCG vaccination consistently protects against a BCG challenge, independent of vaccine dose, route of challenge (intradermal or intranasal) or the interval between vaccination and challenge.^137^ Similar findings have been seen using a novel BCG intranodal challenge model in cattle.^138^ Other murine studies have demonstrated the efficacy of BCG vaccination against *M. tb* aerosol challenge.^139-141^ Taken together, these data suggest that intradermal BCG challenge may reflect a pulmonary vaccine effect, supporting the relevance of a mycobacterial skin challenge in predicting vaccine efficacy against *M. tb.* This approach has most recently been used in a human model in which healthy BCG-naïve and BCG-vaccinated volunteers were challenged with intradermal BCG and BCG load was quantified from skin biopsy specimens. In previously BCG-vaccinated individuals, quantitative PCR analysis of biopsies reflected a degree of mycobacterial immunity to challenge.^142^ These data were supported by subsequent transcriptomic analysis showing that immune signatures, particularly IFN-γ and IL-17 pathways, were strongly induced in previously BCG-vaccinated volunteers and correlated with reduced mycobacterial growth following BCG challenge.^143,144^

However, in early studies culture data were not supportive of the PCR findings in detecting a difference between naïve and vaccinated groups, which may be due to an overestimation of the protective effect of BCG by PCR, or less contemporaneous and reliably comparable results obtained by culture.^142^ In addition, a major limitation to date has been low mycobacterial recovery which has reduced the sensitivity of the model and its ability to discriminate between individuals with differing levels of vaccine-induced anti-mycobacterial immunity. Optimisation studies addressing this issue by evaluating the effect of BCG strain and dose on mycobacterial recovery are in progress. Furthermore, confirmation that BCG challenge reflects pulmonary vaccine effect may ultimately require parallel intradermal and pulmonary challenge trials and comparison of validated immune correlates of protection. Interestingly, a novel intranodal BCG challenge model in cattle has previously shown that BCG vaccine effect is similar to that seen following aerosol challenge with *M. bovis*.^138^ While it is recognized that further optimisation and development of human BCG challenge models is needed, together with consideration of the target population and type of vaccine candidate that the model has greatest relevance to, the concept represents a promising development that warrants further investigation.

There is increasing interest in matching the route of vaccination to the route of natural infection and the first phase I trial assessing a candidate vaccine administered to humans by aerosol has been completed.^145^ The rationale for this approach is that local protective immune responses may be enhanced following delivery of a TB vaccine directly to the respiratory mucosa, thus optimising protection against pulmonary disease. Similarly, the development of a human aerosol BCG challenge model, in a carefully controlled setting, would be more representative of natural infection and could validate an intradermal BCG challenge model.

Finally, in the future there will undoubtedly still be a need to conduct clinical efficacy trials of vaccines that have been highly selected and show greatest potential. Undertaking trials in high TB incidence settings may be of particular value. A study among adolescents in South Africa showed that 50% of the cohort had evidence of LTBI, as demonstrated by a positive IFN-γ release assay (IGRA).^146,147^ It has been estimated that with a 10% annual conversion rate and 0.05% error, a vaccine trial powered to show a 60% effect against infection would need 1000 participants and one year of follow-up.^55^ In contrast to regions with low rates of *M. tb* infection where IGRA serial testing shows frequent conversions and reversions, among adolescents in a high incidence setting there is less IGRA variability, conversion rates are similar to those seen with TST and predict the development of active TB disease in subsequent years.^148-150^ Therefore, IGRAs may have utility as a biomarker for *M. tb* infection in such settings and offer an endpoint for vaccine trials.^55^

However, several of the issues previously discussed that may impact vaccine efficacy in human clinical trials will be prevalent in these settings, such as exposure to NTM and helminth infection.

## Conclusions

TB remains one of the greatest burdens on global health and the importance of an effective vaccine in controlling the epidemic is undisputed. Over the past 20 years, much progress in TB vaccine development has been made resulting in several candidates in clinical assessment. While the results of recent efficacy trials have been disappointing, they have highlighted the need to overcome important obstacles which will be essential if we are to succeed in developing a vaccine against *M. tb*, such as the need to define and validate biosignatures of immune correlates of vaccine-induced protection. This has forced the field to re-evaluate its approach to TB vaccine design and evaluation which has been a positive outcome, complemented by the emergence of exciting and potentially very powerful technologies such as those of systems vaccinology. Despite the many challenges ahead, with continued coordination and collaboration within the TB vaccine community, iterative progress will be made.
